# Diabetes mellitus exacerbates experimental autoimmune myasthenia gravis via modulating both adaptive and innate immunity

**DOI:** 10.1186/s12974-021-02298-6

**Published:** 2021-10-26

**Authors:** Peng Zhang, Chun-Lin Yang, Tong Du, Yu-Dong Liu, Meng-Ru Ge, Heng Li, Ru-Tao Liu, Cong-Cong Wang, Ying-Chun Dou, Rui-Sheng Duan

**Affiliations:** 1 Department of Neurology, The First Affiliated Hospital of Shandong First Medical University & Shandong Provincial Qianfoshan Hospital, No. 16766, Jingshi Road, Jinan, 250014 People’s Republic of China; 2 Shandong Institute of Neuroimmunology, Jinan, 250014 People’s Republic of China; 3 Shandong Key Laboratory for Rheumatic Disease and Translational Medicine, Jinan, 250014 People’s Republic of China; 4 Present Address: School of Medicine, Tongji University, Shanghai, 200092 People’s Republic of China; 5 College of Basic Medical Sciences, Shandong University of Traditional Chinese Medicine, Jinan, 250355 People’s Republic of China

**Keywords:** Diabetes mellitus (DM), Myasthenia gravis (MG), Antibody, Tfh, B cell, Innate immunity

## Abstract

**Background:**

Diabetes mellitus (DM) is a common concomitant disease of late-onset myasthenia gravis (MG). However, the impacts of DM on the progression of late-onset MG were unclear.

**Methods:**

In this study, we examined the immune response in experimental autoimmune myasthenia gravis (EAMG) rats with DM or not. The phenotype and function of the spleen and lymph nodes were determined by flow cytometry. The serum antibodies, Tfh cells, and germinal center B cells were determined by ELISA and flow cytometry. The roles of advanced glycation end products (AGEs) in regulating Tfh cells were further explored in vitro by co-culture assays.

**Results:**

Our results indicated clinical scores of EAMG rats were worse in diabetes rats compared to control, which was due to the increased production of anti-R97–116 antibody and antibody-secreting cells. Furthermore, diabetes induced a significant upregulation of Tfh cells and the subtypes of Tfh1 and Tfh17 cells to provide assistance for antibody production. The total percentages of B cells were increased with an activated statue of improved expression of costimulatory molecules CD80 and CD86. We found CD4^+^ T-cell differentiation was shifted from Treg cells towards Th1/Th17 in the DM+EAMG group compared to the EAMG group. In addition, in innate immunity, diabetic EAMG rats displayed more CXCR5 expression on NK cells. However, the expression of CXCR5 on NKT cells was down-regulated with the increased percentages of NKT cells in the DM+EAMG group. Ex vivo studies further indicated that Tfh cells were upregulated by AGEs instead of hyperglycemia. The upregulation was mediated by the existence of B cells, the mechanism of which might be attributed the elevated molecule CD40 on B cells.

**Conclusions:**

Diabetes promoted both adaptive and innate immunity and exacerbated clinical symptoms in EAMG rats. Considering the effect of diabetes, therapy in reducing blood glucose levels in MG patients might improve clinical efficacy through suppressing the both innate and adaptive immune responses. Additional studies are needed to confirm the effect of glucose or AGEs reduction to seek treatment for MG.

## Background

Diabetes mellitus (DM) is a chronic metabolic disorder caused by insulin deficiency or impaired insulin action, which is characterized by increased blood glucose levels (hyperglycemia). There are three well-accepted categories of DM, Type 1 diabetes (T1DM), Type 2 diabetes (T2DM), and gestational diabetes mellitus (GDM) [1]. Inflammatory and immunologic abnormalities are involved in both T1DM and T2DM, as well as their associated complications [2]. Although the accurate mechanisms remain to be fully clarified, functional alterations of the immune system and related cells could, at least in part, be attributed to hyperglycemia. Patients with hyperglycemia have increased pro-inflammatory cytokines and dysfunctional innate and adaptive immune cells, which will disrupt cellular and/or humoral immune homeostasis [2]. Hyperglycemia apparently alters the functions of immune cells, such as monocytes, neutrophils, and T or B lymphocytes [3]. In addition, T regulatory cells obtained from diabetes patients and streptozotocin (STZ)-induced diabetic mice displayed defective immunosuppressive functions [2, 4]. Prolonged hyperglycemia has been reported to be associated with increased production of advanced glycation end products (AGEs), which triggered inflammation through binding specific receptors. AGEs induced the maturation of dendritic cells (DCs) and augmented their capacity to stimulate the proliferation and cytokine production of T cells [5]. In addition, AGEs promote the differentiation of CD4^+^ T into T helper 1 (Th1) and Th17 cells [6].

Myasthenia gravis (MG) is an antibody-mediated, T-cell-dependent autoimmune disease. Antibodies targeting neuromuscular junction molecular impaired the neuromuscular transmission and lead to fatigable muscle weakness. Abnormal Th1, Th17, and follicular helper T (Tfh) cells have been shown play critical roles in the pathogenesis of MG by promoting auto-reactive antibody generation [7–9]. It is reported diabetes was more prevalent with late-onset myasthenia gravis (LOMG) [10]. Many patients had been suffering from diabetes when they were diagnosed with MG. Few studies focused on the effect of DM and hyperglycemia on the incidence and severity of MG. In this study, we investigated the influence of DM on the both adaptive and innate immune systems in a well-established EAMG animal model. Exploring the overlapping roles of diabetes in MG will facilitate the further understanding in the pathogenesis of MG patients with DM and may provide more insights into the importance of clinical blood glucose level control in immune regulation.

## Materials and methods

### Animals and experimental design

Female Lewis rats, 6–8 weeks, weighing 150–170 g (Vital River Corporation. Beijing, China) were used for the study. The animals were housed in polypropylene cages in a clean room with a 12 h light/dark cycle, with free access to standard laboratory chow and water. Animals were divided into two groups, EAMG with or without DM (*n* = 8 in the DM+EAMG group and *n* = 7 in the EAMG group). Hyperglycemia was induced in overnight fasted rats with a single intraperitoneal (i.p.) administration of streptozotocin (STZ, 60 mg/kg, Sigma-Aldrich, St. Louis, MO, USA). STZ was dissolved in a citrate acid buffer solution (0.1 M citric acid and 0.1 M sodium citrate). Age and weight-matched rats in the control group received an equivalent volume of citrate acid buffer solution alone. Hyperglycemia (blood glucose > 200 mg/dl) was confirmed 3 day post-STZ injection using a glucometer (AccuCheck; Roche, Germany). Four days after STZ injection, rats from both diabetic and control groups were immunized with 75 µg of R 97–116 peptide emulsified in CFA subcutaneously (peptide from China Peptides Co., Ltd.; Shanghai, China), and boosted with 75 µg of R97–116 peptide emulsified in IFA in 200 μl injected at the tail base 30 days later. Before immunization (day 0) and thereafter every other day, body weights and clinical scores were recorded and assessed in a blinded fashion as follows: 0, normal strength and no abnormalities; 1, mildly decreased activity and weak grip or cry, more evident at the end of exercise; 2, clinical signs present before exercise (tremor, head down, hunched posture, weak grip); 3, severe clinical signs present before exercise, no grip, moribund; 4, dead.

### Measurement of spleen weight and flow cytometry analysis of spleens and lymph nodes

On day 50 (peak of the disease) after induction of EAMG, rats were euthanized under deepening anesthesia. Spleens and inguinal lymph nodes were removed and the weights were measured. Then the spleens and inguinal lymph nodes were minced through a 70 μm cell strainer to prepare single-cell suspensions of mononuclear cells (MNCs) under aseptic conditions. Red blood cells in spleen MNCs were lysed with a lysis buffer (Biolegend) for 5 min. All specimens were coded to facilitate blind testing. The spleen or inguinal lymph nodes MNCs suspension with a final volume of 100 μl were labeled with corresponding monoclonal antibodies and incubated for 30 min at 4 °C in the dark. The following antibodies were used in the assay: CD3 (1F4; Biolegend), CD4 (OX35; eBioscience), B220 (HIS24; eBioscience), CD20 (SP32; Abcam), CD161 (10/78; BD Pharmingen), MHC II (HIS19; Biolegend), CD80 (3H5; Biolegend), CD86 (24F; Biolegend), CD40 (HM40-3; Biolegend), CD27 (LG.7F9; eBioscience), ICOS (C398.4A; Biolegend), CXCR5 (ERP8837; Abcam). Unconjugated primary antibodies were detected with Alexa Fluro 488-conjugated anti-rabbit IgG (Abcam). Samples were analyzed using an Aria II flow cytometer (BD).

### Intracellular cytokine and antibody-secreting cells analysis

For intracellular cytokine staining, cells were stimulated for 5 h with a cell stimulation cocktail (eBioscience), followed by surface staining with CD4 for 30 min at 4 °C. To stain intracellular antigens and antibodies, cells were permeabilized with IC Fixation Buffer and Permeabilization Buffer (eBioscience) following the manufacturer’s instruction and stained with anti-IFN-γ (DB-1; Invitrogen), anti-IL-17A (eBio17B7; eBioscience), anti-IL-4 (OX81; eBioscience), anti-IgG (Poly4054; Biolegend) or anti-Ig light chain κ (MRG81; Biolegend), respectively. Stained cells were analyzed with BD AriaIIflow cytometer and Flowjo software (TreeStar Inc., Ashland, OR, USA).

For regulatory T (Treg) cells staining, cells were stained with surface marker CD4 (OX35; eBioscience) and CD25 (OX39; Invitrogen) before being fixed and permeabilized with Perm/Fix solution (eBioscience). Finally, the antibody for Foxp3 (FJK-16S; eBioscience) was added for 30 min in the dark. Stained cells were analyzed with a flow cytometer and Flowjo software.

### Isolation of T, B, and NK cells and co-culture assays in vitro

Splenic T cells, B cells, and natural killer (NK) cells were purified using the method previously described [11]. In brief, the rat spleen was obtained and splenocytes were isolated after lysing red blood cells using RBC lysis buffer. CD3^+^ T cells, B220^+^ B cells, and CD3^−^CD161^+^ NK cells were prepared using magnetic beads according to the manufacturer’s instructions, respectively. To purify T cells, splenocytes were stained with Alexa Fluor 647-conjugated anti-CD3 (1F4; Biolegend) and then incubated with anti-Cy5/anti-Alexa Fluor 647 Microbeads (Miltenyi Biotec). Positive selection was performed using an OctoMACS™ Separator (Miltenyi Biotec). To purify B cells, splenocytes were stained with PE-conjugated anti-B220 (HIS24; eBioscience) and then incubated with anti-PE Microbeads (Miltenyi Biotec). Positive selection was performed to obtain B cells. The isolation of NK cells was performed in two steps. Splenocytes were stained with Alexa Fluor 647-conjugated anti-CD3 (1F4; Biolegend) and PE-conjugated anti-CD161(10/78; BD Pharmingen). Then stained cells were incubated with Anti-Cy5/Anti-Alexa Fluor 647 Microbeads (Miltenyi Biotec). The negative selection was performed to deplete the CD3-positive cells. Finally, the flow-through fraction was incubated with anti-PE microbeads. Positive selection was performed to obtain the NK cells. DCs were obtained from bone marrow as previously reported [12].

The activation of T cells was performed using purified mouse anti-rat CD3 (eBioG4.18; eBioscience) and anti-CD28 antibodies (JJ319, BD Bioscience). The plates were pre-coated with anti-CD3 antibodies (1 μg/ml) overnight at 4 °C. After washing the wells with PBS, T cells were seeded in the presence of soluble anti-CD28 antibodies (1 μg/ml).

T-cell differentiation experiments were performed as the following. (i) Splenocytes or purified T cells were re-suspended to 2 × 10^6^/ml and seeded into 24-well round-bottom cell culture plates and incubated with different concentrations of glucose or AGEs (Abcam). After 72 h of culture, cells were collected, stained for Th1, Th2, Th17, Treg, and Tfh cells, and analyzed using flow cytometry. (ii) Purified T cells were co-cultured with purified B cells, NK cells, BMDCs or not (T: NK/B/DC = 1:1), respectively. After 72 h, the cells were harvested, stained with corresponding antibodies, and detected by the flow cytometry.

The activation of B cells was performed using LPS (Sigma-Aldrich). B cells were incubated with PBS or 200 μg/ml AGEs for 48 h and the surface marker was analyzed using flow cytometry.

### Serological evaluation of peptide R97–116-specific Ab production

To evaluate Ab production, 96-well microtiter plates (Costar) were coated with 10 µg/ml of peptide R97–116. Plates were incubated with serum samples diluted 1:100, with bound antibody detected using biotin goat anti-rat IgG, IgG1, IgG2a, or IgG2b antibodies (all from Biolegend), respectively.

### Statistical analysis

Data were expressed as mean ± SEM. GraphPad Prism version 8.0 for Windows (GraphPad Software, USA) was used for the statistical analysis. The Student’s *t* test for unpaired data was used for comparisons between groups. *p* Values < 0.05 were considered statistically significant.

## Results

### Diabetes exacerbated the clinical severity and reduced the splenic volume in EAMG rats

**Fig. 1:**
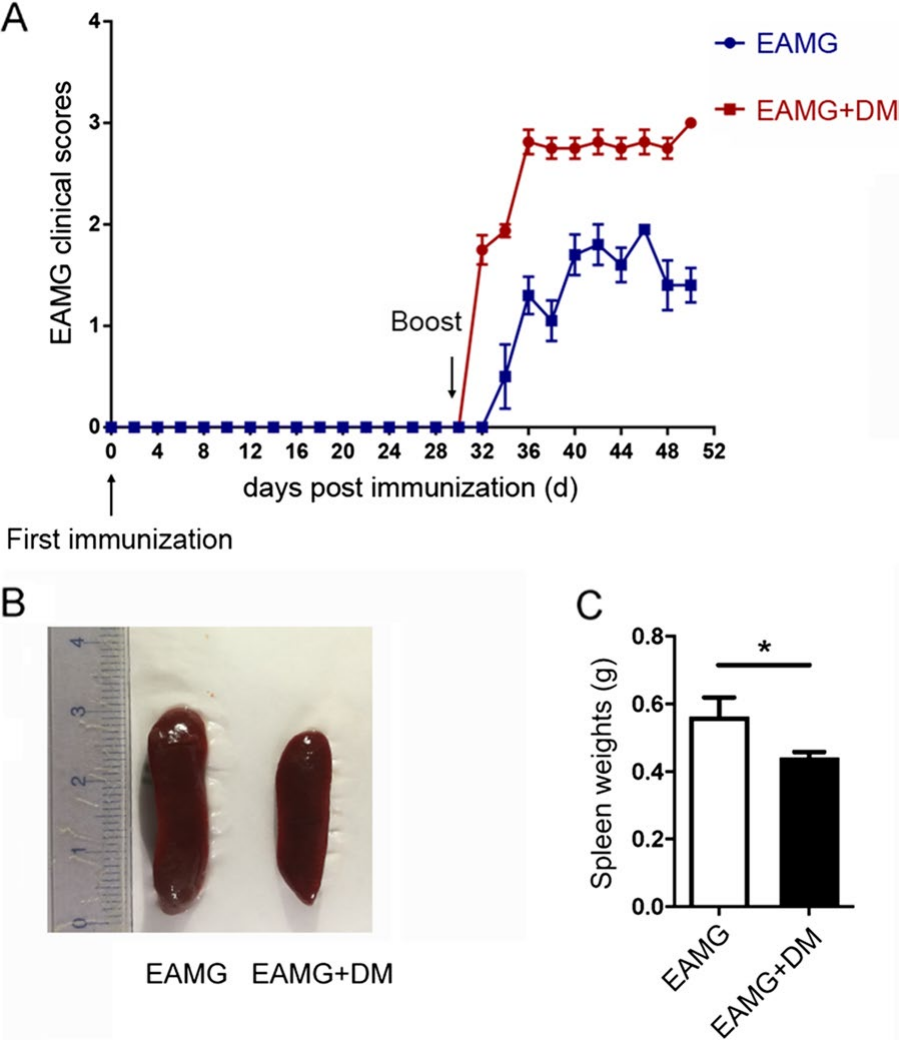
Clinical scores and the splenic volume of EAMG in rats with or without DM. **A** EAMG model was induced in DM or control rats and the clinical scores were record every 2 days until day 50 p.i. On day 50, **B** examples of spleens from different groups are provided, and the weights of the spleen were measured after euthanizing the rats (**C**). Data were from two independent experiments and expressed as mean ± SEM. *n* = 8 in the DM+EAMG group and *n* = 7 in the EAMG group. The significance of differences was assessed by the Unpaired Student’s *t* test. ns means not significant, *p < 0.05, **p < 0.01 and ***p < 0.01

Lewis rats were divided into two groups, control and DM. For the DM groups, rats were treated with a single dosage of STZ, while the rats in the control groups were received an equivalent volume of citrate acid buffer solution alone. The efficiency of DM induction was verified by measuring blood glucose (BG) levels 3 days later (Table 1). Then rats from both groups were immunized with two doses of peptide R97–116 with a dose interval of 30 days. As shown in Fig. 1, rats in the DM+EAMG group showed earlier onset and more severe clinical symptoms compared with EAMG rats. After disease onset, the progression of clinical scores appeared to advance more rapidly in the DM+EAMG group compared to the EAMG group, and the differences were statistically significant on days 38, 42, 44, 46, 48, and 50 p.i.. At the end of the experiment, the mean clinical score was 1.813 in the DM+EAMG group and 1.107 in the EAMG group. To observe the effect of diabetes on immune organs more intensively, rats were sacrificed and the spleens were weighed on day 50 p.i. The results showed that diabetes led to significant reductions in splenic weights (Fig. 1B, C).

**Table 1:** BG level of rats in the two groups

Post STZ injection	Day 3			Day 13			Day 53		
	DM+EAMG	EAMG	*p* value	DM+EAMG	EAMG	*p* value	DM+EAMG	EAMG	*p* value
BG (mg/dl)	449.8 ± 26.2	122.1 ± 4.4	0.001	451.3 ± 21.2	119.8 ± 6.7	0.001	549.0 ± 12.7	105.4 ± 3.9	0.001

Values are mean ± SEM. *n* = 8 in the DM+EAMG group and *n* = 7 in the EAMG group. Unpaired Student’s *t* test was used

*BG* blood glucose

### The levels of anti-R97–116 peptide antibodies are higher in EAMG rats with DM, along with the increased frequencies of antibody-secreting cells and memory B cells

**Fig. 2:**
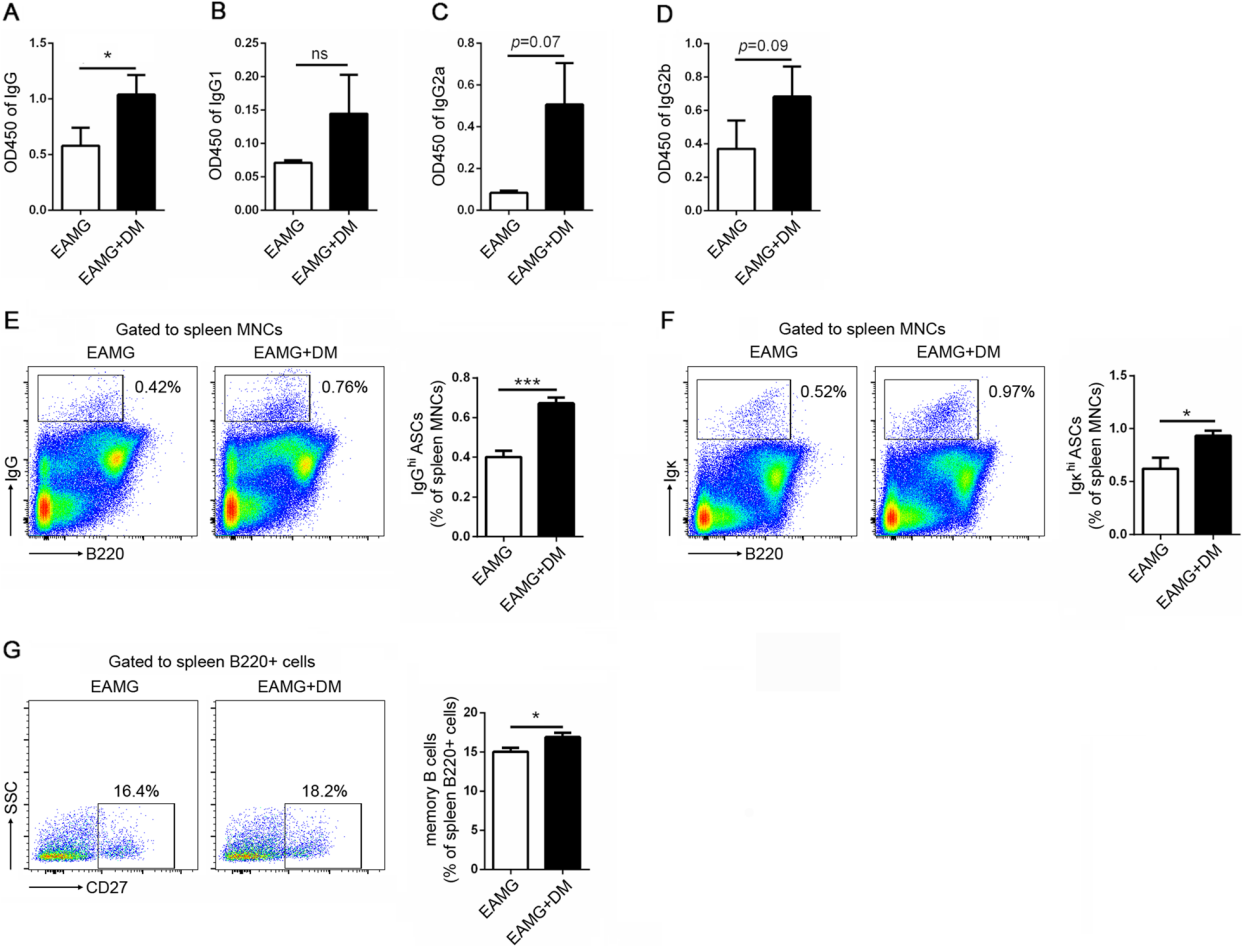
Effects of diabetes on the antibody production and B cells differentiation in EAMG rats. Serum anti-R97–116 IgG (**A**), IgG1 (**B**), IgG2a (**C**), and IgG2b (**D**) were determined by ELISA. Percentages of IgG^hi^ ASCs (**E**), Igκ^hi^ ASCs (**F**), and memory B cells (**G**) were assessed by flow cytometry and representative dot plot were shown, respectively. Data were from two independent experiments and expressed as mean ± SEM. *n* = 8 in the DM+EAMG group and *n* = 7 in the EAMG group. The significance of differences was assessed by the Unpaired Student’s *t* test. ns means not significant, *p < 0.05 and ***p < 0.01

Myasthenia gravis is characterized by an increase in the production of auto-reactive antibodies, which are known to be pathogenic. To examine the effect of DM on the antibody generation in EAMG rats, sera in both groups were collected and the levels of antibodies were measured by ELISA. As shown in Fig. 2A, the levels of anti-R97–116 IgG were significantly higher in the DM+EAMG group compared to the EAMG group. However, there were no differences in the subtypes of anti-R97–116 IgG, including IgG1, IgG2a, and IgG2b (Fig. 2B–D). Moreover, since antibody‐secreting cells (ASCs) are responsible for the production of antibodies, flow cytometric analysis was performed and the results revealed that there were significantly more ASCs (Fig. 2E, F), which is defined as B220^−^IgG^hi^ or B220^−^Igκ^hi^ in the spleens of rats in the DM+EAMG group compared to the EAMG group.

To further evaluate the mature B cell differentiation, the percentages of memory B cells (defined as B220^+^CD27^+^) were also examined by flow cytometry. It was shown that the percentages of memory B cells were upregulated in the DM+EAMG group (Fig. 2G).

### Diabetes shaped immune balance towards a pro-inflammatory phenotype in EAMG rats

**Fig. 3:**
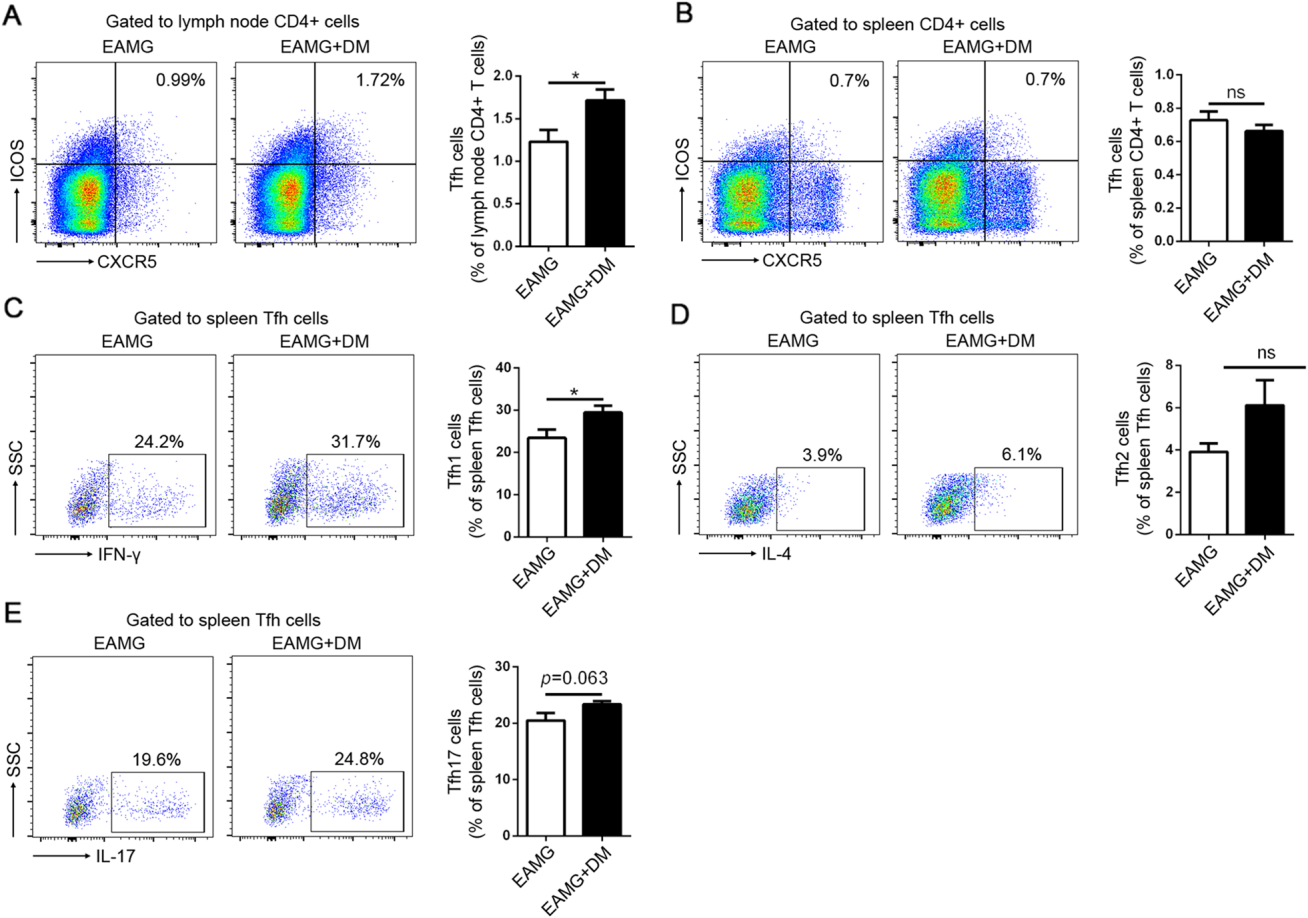
Effects of diabetes on Tfh cells and the subtypes. The percentages of Tfh cells in the lymph nodes were assessed (**A**). The percentages of total Tfh cells (**B**), Tfh1 (**C**), Tfh2 (**D**), and Tfh17 (**E**) among spleen MNCs were analyzed by flow cytometry. Data were from two independent experiments and expressed as mean ± SEM. *n* = 8 in the DM+EAMG group and *n* = 7 in the EAMG group. The significance of differences was assessed by the Unpaired Student’s *t* test. ns means not significant, *p < 0.05

Differentiated CD4^+^ T cells are divided into different subtypes based on the cytokines they synthesize and secret, which include Th1, Th2, Th17, and Treg cells. The imbalance of T-cell subtypes is shown to have a fundamental role in the pathogenesis of MG [13–16]. Augment of Th1 and Th17 cells and the impaired function of Treg cells result in increased levels of pro-inflammatory cytokines, which perpetuate the immune dysregulation in MG [17]. Th1 (CD4^+^IFN-γ^+^), Th2 (CD4^+^IL-4^+^), Th17 (CD4^+^IL-17^+^), and Treg cells (CD4^+^CD25^+^Foxp3^+^) were analyzed by FACS, respectively. As shown in Fig. 3, diabetes upregulated percentages of Th1 cells and down-regulated Treg cells in the EAMG model. However, there were no differences in Th17 cells between the two groups. These results indicated that diabetes promoted the phenotype of pro-inflammatory T cells.

### Diabetes increased Tfh cells in lymph nodes and upregulated Tfh1 and Tfh17 cells in the spleen of EAMG rats

**Fig. 4:**
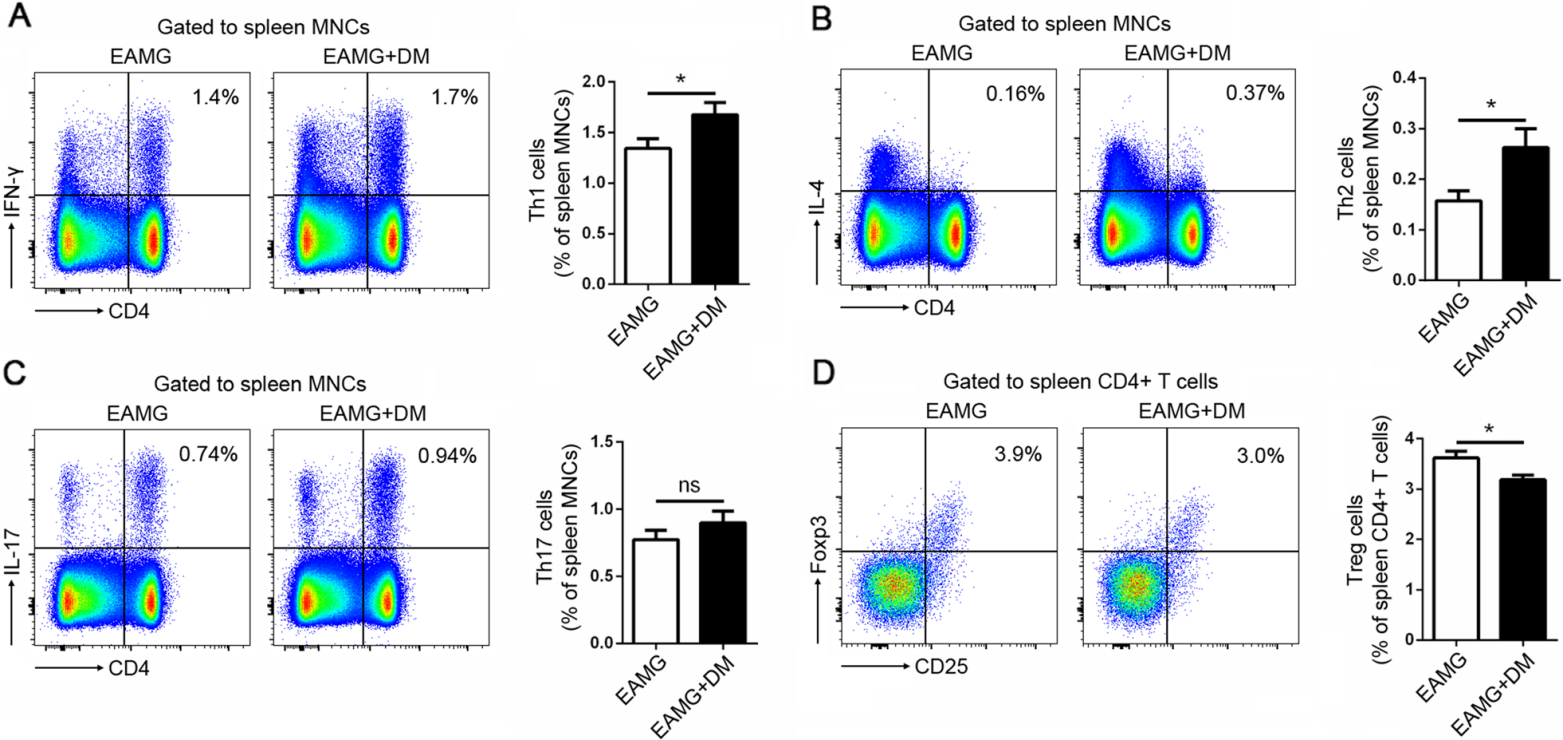
Effects of diabetes on the T helper cell differentiation in EAMG rats. The percentages of Th1 (**A**), Th2 (**B**), Th17 (**C**), and Treg cells (**D**) among spleen MNCs were analyzed by flow cytometry. Data were from two independent experiments and expressed as mean ± SEM. *n* = 8 in the DM+EAMG group and *n* = 7 in the EAMG group. The significance of differences was assessed by the Unpaired Student’s *t* test. ns means not significant, *p < 0.05

MG is an antibody-mediated autoimmune disease. The antibody generation requires the collaboration of multiple cell types, in which T follicular helper (Tfh) cells provide critical and specialized help to germinal center (GC) B cells through cognate T–B cell interactions [18]. According to the cytokine profiles, Tfh cells could be divided into Tfh1 (produce IFN-γ), Tfh2 (produce IL-4), and Tfh 17 (produce IL-17) cells, which play different roles in shaping humoral immune response and contributing to the development of MG [9, 19]. Therefore, we detected the percentages of Tfh cells and the subtypes in both groups. As shown in Fig. 4A, the percentages of Tfh cells in the lymph nodes were enhanced significantly in the DM+EAMG group compared to the EAMG group. The upregulated Tfh cells were in accordance with the increased antibody level, antibody-secreting cells, and memory B cells. However, the change in Tfh was not observed in the spleen. Furthermore, the subtypes of Tfh cells in the spleen, based on the cytokine secretion, were also examined by FACS. Results showed that both Tfh1 (Fig. 4C) and Tfh17 cells (Fig. 4E) were raised in the DM+EAMG group. There was no statically significant difference regarding Tfh2 cells between the two groups (Fig. 4D).

### B cells activation was increased in the DM+EAMG group

**Fig. 5:**
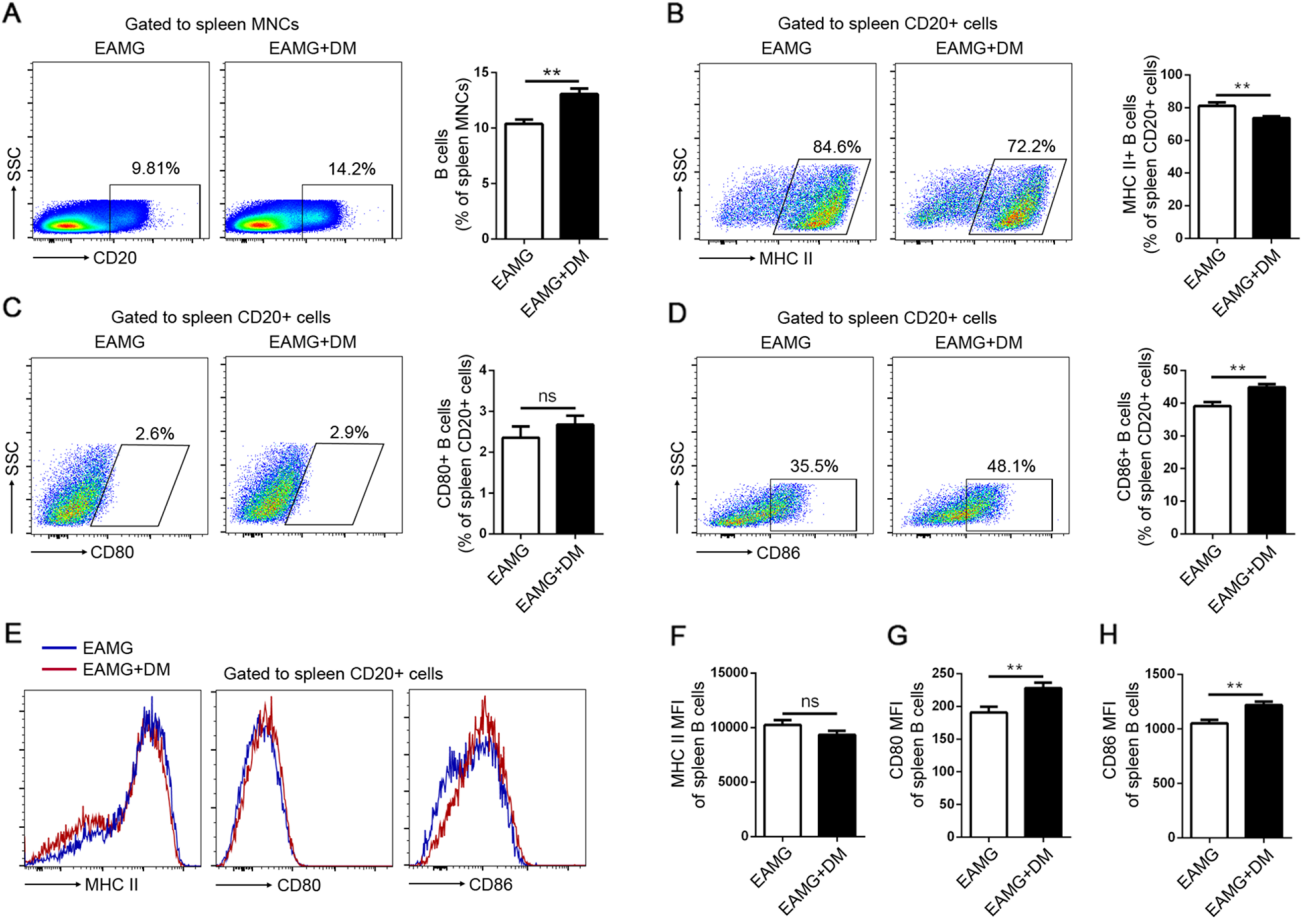
Expression of MHCII, CD80, and CD86 on B cells. The percentages of total CD20^+^ B cells (**A**), MHCII (**B**), CD80 (**C**), and CD86 (**D**) on CD20^+^ B cells were assessed by flow cytometry. **E** Representative data of MFI were shown. MFI analysis of MHCII (**F**), CD80 (**G**), and CD86 (**H**) were analyzed. Data were from two independent experiments and expressed as mean ± SEM. *n* = 8 in the DM+EAMG group and *n* = 7 in the EAMG group. The significance of differences was assessed by the Unpaired Student’s *t* test. ns means not significant, **p < 0.01

Tfh and B cell interactions are critical not only for B cell survival and activation but also for Tfh differentiation. Signals from cognate B cells are necessary for the complete differentiation and survival of Tfh cells in the germinal center [20]. The percentages of CD20^+^ B cells were upregulated in the DM+EAMG group compared to the EAMG group (Fig. 5A). Thus, we further evaluated whether the activation state of B cells was influenced by diabetes. As shown in Fig. 5B–D, the percentages of MHC II were decreased but the percentages of CD86 were increased in the DM+EAMG group. We also analyzed the mean fluorescence intensity (MFI) of these molecules and found that diabetes enhanced the MFI of CD80 (Fig. 5G) and CD86 (Fig. 5H) on B cells in EAMG rats. These results indicated that diabetes stimulated the activation of B cells in EAMG.

### Effect of diabetes in innate immunity of EAMG rats

**Fig. 6:**
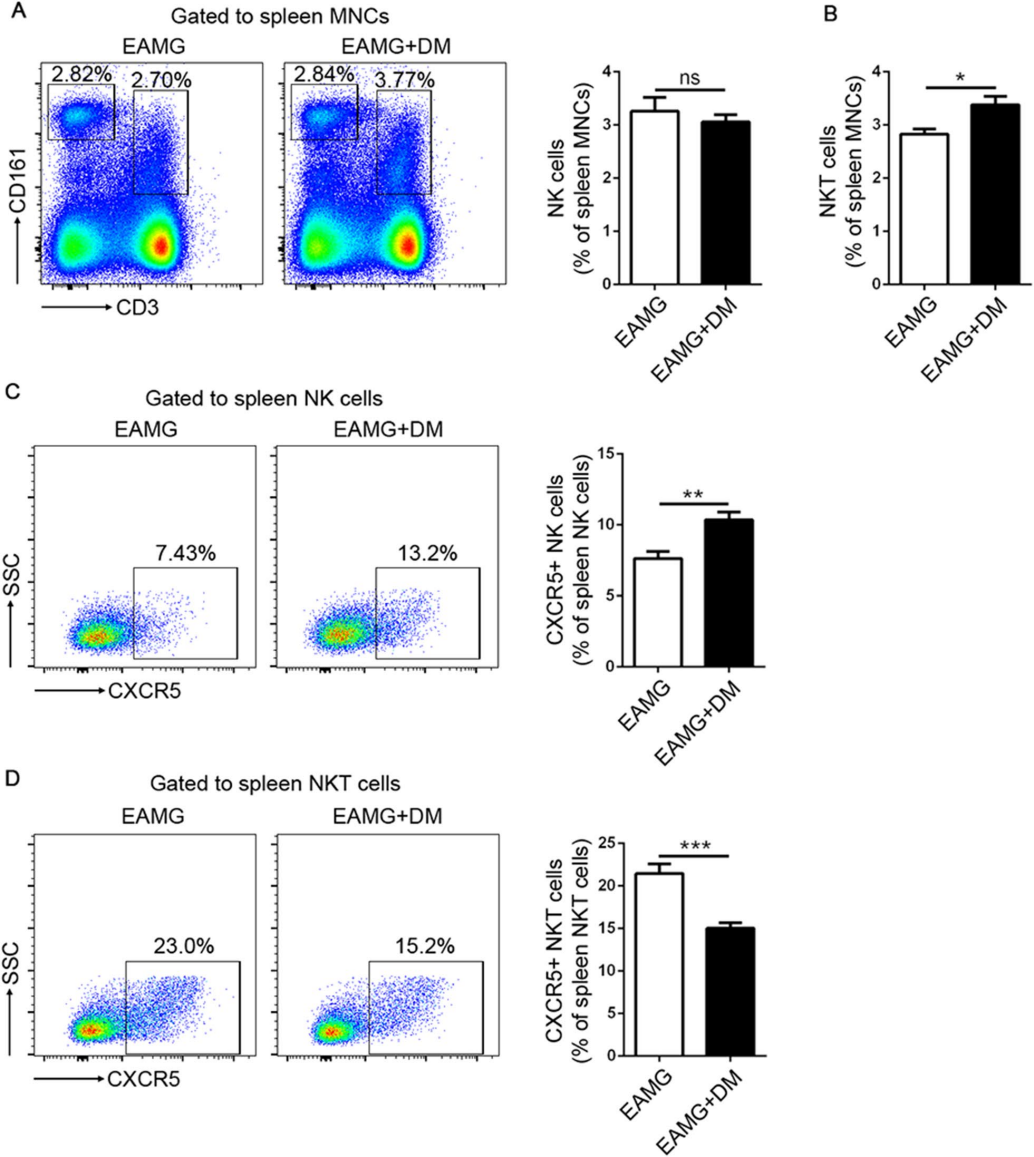
Effects of diabetes on NK and NKT cells frequency and the expression of CXCR5 on these cell types. Percentages of NK cells (**A**) and NKT cells (**B**) among spleen MNCs were shown. Expression of CXCR5 on NK cells (**C**) and NKT cells (**D**) was shown. Data were from two independent experiments and expressed as mean ± SEM. The significance of differences was assessed by the Unpaired Student’s *t* test. *n* = 8 in the DM+EAMG group and *n* = 7 in the EAMG group. ns means not significant, *p < 0.05, **p < 0.01 and ***p < 0.01

To address the effect of diabetes on the innate immunity of EAMG rats, we analyzed the percentages of NK cells and natural killer T (NKT) cells. NK and NKT cells were defined as CD3^−^CD161^hi^ and CD3^+^CD161^+^, respectively. Flow cytometry results showed that the percentages of NKT cells were downregulated in the DM+EAMG group compared to the EAMG group. However, there was no change in the percentages of NK cells. Furthermore, we tested the expression of CXCR5 on NK and NKT cells and found an increase of CXCR5 expression on NK cells (Fig. 6C), whereas a decreased CXCR5 expression on NKT cells (Fig. 6D) in the DM+EAMG group.

### Enhanced Tfh differentiation was mediated by AGEs but not hyperglycemia in vitro

**Fig. 7:**
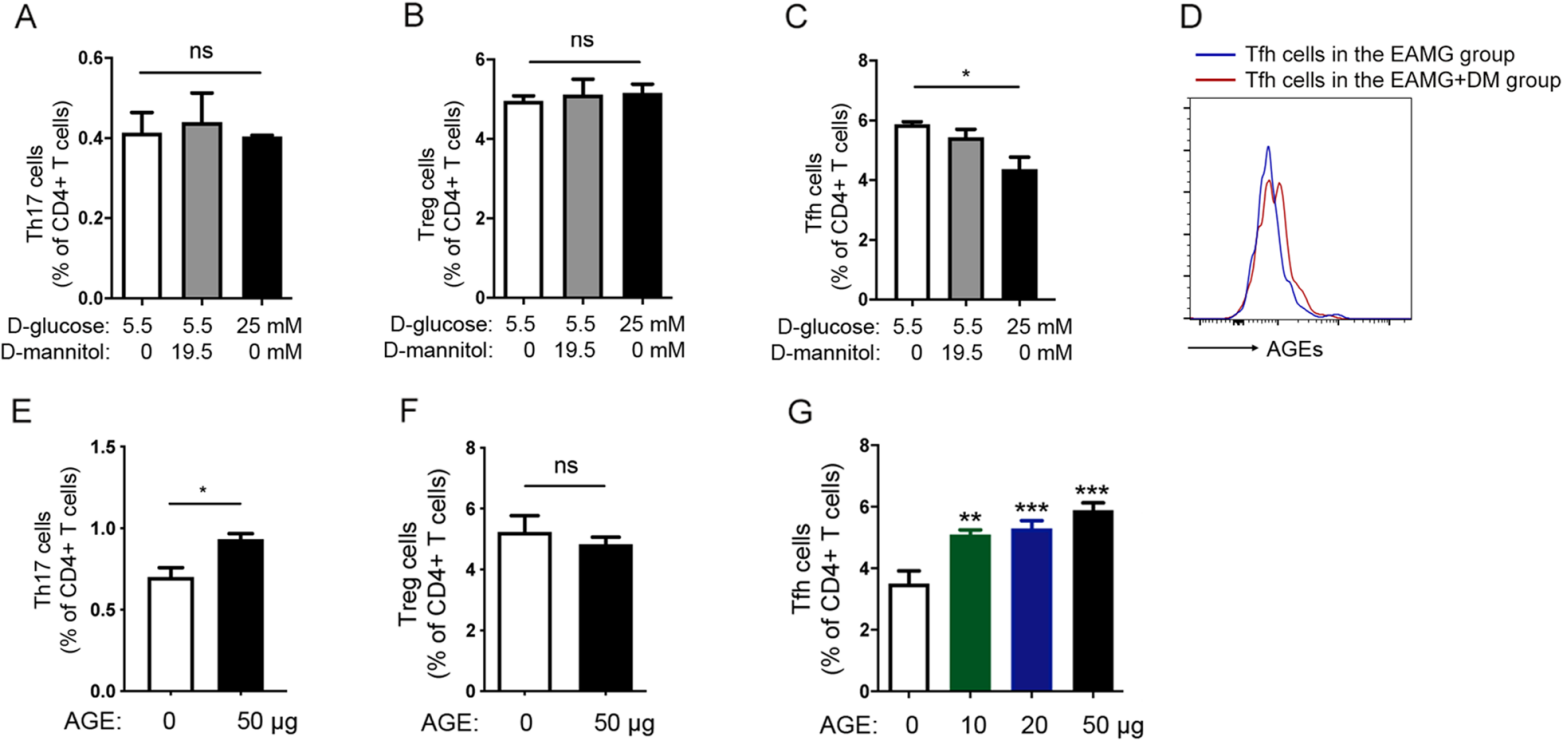
Effects of d-glucose and AGEs on differentiation of splenic CD4^+^ T cells in vitro. Splenocytes were cultured with different concentrations of d-glucose and d-mannitol and the percentages of Th17 (**A**), Treg cells (**B**), and Tfh cells (**C**) were analyzed by flow cytometry. **D** The AGEs MFI in splenic Tfh cells from both EAMG rats and EAMG rats with DM were analyzed by flow cytometry. Then splenocytes were cultured with AGE and the percentages of Th17 (**E**) and Treg (**F**) were analyzed by flow cytometry. **G** Splenocytes were treated with different concentrations of AGEs and Tfh cells were examined by flow cytometry. Data were from two independent experiments and expressed as mean ± SEM. The significance of differences was assessed by the Unpaired Student’s *t* test or ANOVA, followed by Least Significant Difference (LSD) as a post-hoc test. ns means not significant, *p < 0.05, **p < 0.01 and ***p < 0.001

To further explore the mechanisms of the enhanced immune response in the EAMG rats with diabetes, we first cultured the spleen MNCs in high glucose conditions. To nullify the osmotic stress caused by the high concentration of solute in the culture medium, d-mannitol was added in the culture medium. However, neither low-level nor high-level concentration of d-glucose boosted the differentiation of CD4^+^ T cells towards Th17, Treg, or Tfh cells (Fig. 7A–C). Studies have shown that the high level of AGEs may partially account for the pro-inflammatory conditions of DM. Indeed, Tfh cells from EAMG+DM rats exhibited increased intracellular AGE accumulation compared to EAMG rats, as evidenced by upregulated AGEs mean fluorescent intensity (MFI) in Tfh cells (Fig. 7D). To further test the effect of AGEs on T-cell differentiation, spleen MNCs were treated with or without AGEs. Results showed an increase in the percentages of Th17 cells but no change of the Treg cells in the AGEs-treated group when compared to the control group (Fig. 7E, F). Furthermore, the percentages of Tfh cells among CD4^+^ T cells were also increased by the treatment of AGEs and in a dose-dependent manner (Fig. 7G).

### AGEs promoted the development of Tfh cells by B cells in vitro

**Fig. 8:**
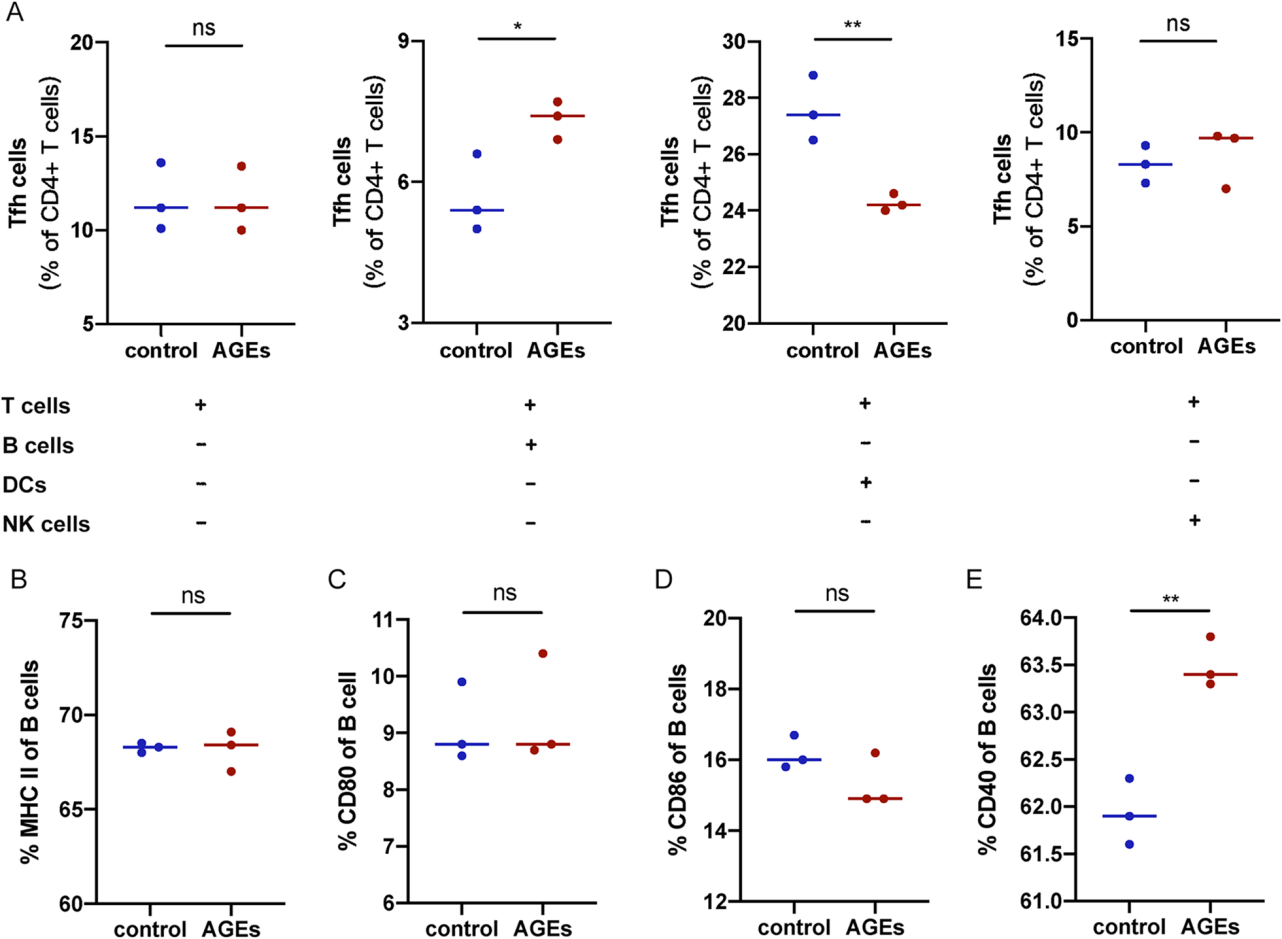
B cells promoted the differentiation of Tfh cells induced by AGEs in vitro. AGEs were added to the culture of purified T cells only or with B cells, NK cells, or DCs for 72 h, respectively. The percentages of Tfh cells were analyzed by flow cytometry (**A**). B cells were cultured with AGEs for 48 h and the phenotype referred to MHC II (**B**), CD80 (**C**), CD86 (**D**), and CD40 (**E**) were calculated by flow cytometry. Data were from two independent experiments and expressed as mean ± SEM. The significance of differences was assessed by the Unpaired Student’s *t* test. ns means not significant, **p < 0.01 and ***p < 0.01

To figure out the mechanism of how AGEs induced the differentiation of Tfh cells, purified splenic CD3^+^ T cells were cultured with AGEs and the percentages of Tfh cells were measured 72 h later. As shown in Fig. 8A, there was no difference in the percentages of Tfh cells between various concentrations of AGEs, which indicates that AGEs may not directly affect Tfh cells differentiation. Next, we further determined which component of MNCs played a vital role in Tfh cells induction in vitro. B cells, NK cells, and bone-marrow-derived DCs were co-cultured with T cells, respectively. Results demonstrated that AGEs could specifically promote the differentiation of Tfh cells in the presence of B cells. However, no evidence exists for an analogous role of DCs and NK cells. These results indicated that the induction of Tfh cells by AGEs depends on B cells.

B cell plays a vital role in the regulation of Tfh cell generation. To further explore the effects of AGEs on B cells, isolated splenic B cells were treated with AGEs in vitro and the surface markers on B cells were measured. A slight but statistically significant increase of CD40 on B cells was observed in the AGEs-treated group. However, there were no significant differences in the expression of MHC II, CD80, and CD86 between the control and the AGEs-treated group (Fig. 8B). These results indicated AGEs increased the CD40 expression on B cells, which may contribute to Tfh cell promotion.

## Discussion

Diabetes mellitus is a chronic multifactorial disease characterized by chronic low-grade inflammation [21]. Many patients had been suffering from diabetes when they were diagnosed with MG. However, few studies focused on the effect of diabetes mellitus and hyperglycemia on the severity of MG. It is reported that Non-obese T2DM patients presented a high level of fecal IgG, which is produced by B cells, compared to body mass index (BMI)-matched controls [22]. Augmentation of immunoglobulin (IgM, IgA, and IgG) production were also found in a rat model of T2DM [23]. In the study, we evaluate how immune responses in EAMG rats were affected by the existence of diabetes and found that diabetes could exacerbate the clinical symptoms of EAMG rats. The elevated production of the elevated anti-R97–116 peptide IgG antibody in DM rats may be the key role to aggravate EAMG, as they directly bind to the AchR at the neuromuscular junction.

Antigen-specific CD4^+^ T cells are essential in the pathogenesis of MG. Based on phenotype and function differences, CD4^+^ T cells can be further divided into Th1, Th2, Th17, Tfh, and Treg cells. Th1 and Th17 cells, which mainly secret the pro‐inflammatory cytokines IFN‐γ and IL-17, respectively [24], play critical roles in both T-cell-mediated and Ab‐mediated autoimmunity, including MG [25]. Th2 cells, which produced interleukin-4 (IL-4), are more effective in stimulation of humoral immune system [26]. A significant increase in the IL-4 expression was observed in T1DM patients compared to controls [27]. Treg cells, define as CD4^+^CD25^+^Foxp3^+^, exert their immunosuppressive effect in various autoimmune diseases. Plenty of studies has demonstrated a decrease of Treg cells in diabetic patients or rodent models compared with healthy controls, which suggests a deficiency of suppressive regulation in the immune system [28]. Our results demonstrated diabetes augments Th1 and Th17 response in EAMG rats, which is in line with previous observations that CD4^+^ T-cell-derived IFN‐γ and IL-17 were increased in diabetic settings both in human and animal models. The reduced percentages of Treg cells in the spleen were also found in EAMG rats with DM. The increased Th1 and Th17 cells and decreased Treg cells in diabetic EAMG rats indicating the immune balance were skewed into pro-inflammatory phenotype by diabetes.

The immune-pathogenic roles of Tfh cells in antibody-mediated autoimmune diseases have been investigated extensively including MG. Tfh cells are a subset of CD4^+^ T helper cells specialized to facilitate the development of humoral immunity [29]. Tfh cells provide signals to GC B cells, which are essential for their survival, differentiation, affinity maturation, isotype switching, and high-affinity antibody secretion. Abnormal high levels of circulating Tfh cells in peripheral blood were observed in both T1DM and T2DM patients [30, 31]. Several reports have observed an increase of Tfh cells in EAMG models and addressed the pathogenic role of Tfh cells in antibody production [32, 33]. In addition, based on the various cytokines they produced, such as IFN‐γ, IL‐4, IL‐10, and IL‐17, Tfh cells are divided into many subtypes. The activated Tfh1 and Tfh17 in Tfh cells are the major source for IL-21 production to affect the disease development in MG patients [9]. Here we found an enhanced level of Tfh cells as well as Tfh1 and Tfh17 cells in the DM+EAMG group, along with the higher clinical scores, anti-R97–116 IgG, and antibody-secreting cells, suggesting the more activated humoral immune state in the DM+EAMG group.

Long-term hyperglycemia can lead to the formation of AGEs. In the STZ-diabetic rodent model, peripheral AGE levels were significantly increased [34]. Accumulation of AGEs induces inflammation and elevated ROS concentration, which ultimately affects cell metabolic activities. These factors further aggravate the accumulation of AGEs, thereby promoting the occurrence and development of diabetes mellitus [35]. When co-cultured with human peripheral blood CD3^+^ T cells, AGEs significantly up-regulated the expression level of ICOS/ICOSL on T cells [36]. In addition, AGEs bind to a specific receptor of advanced glycation end products (RAGE) to trigger an inflammatory response [37, 38], which lead to the development and progression of a variety of inflammatory diseases, including diabetic vascular complications [39], cardiovascular disease (CVD) [40], cancer [41], Alzheimer’s disease (AD) [42]. Previous studies demonstrated that AGEs deposits were upregulated in patients with DM [43], and the AGEs–RAGE signaling pathway could aggravate the clinical symptoms of EAMG rats and promote the proliferation of antibody-specific T cells [44]. However, the relationship between AGEs and Tfh is unclear. In our study, the level of AGEs in Tfh cells of EAMG+DM rats was higher than that of EAMG rats. Furthermore, our ex vivo data showed there were higher percentages of Tfh cells when spleen MNCs were treated with AGEs. However, no differences were observed in Treg, Th17, and Tfh cells when co-cultured with or without d-glucose. These results provided strong evidence that the enhanced immune response in the DM+EAMG group was mediated by AGEs rather than the high-glucose environment. Further experiments illustrated AGEs could not directly induce the differentiation of Tfh cells, and this effect is dependent on the existence of B cells, which leads us to further focus on the regulatory effect of AGEs on B cells.

Tfh cell differentiation is a tightly regulated process. After recognizing cognate antigen present by DCs, naïve T cells initially up-regulate CXCR5 expression and migrate to the T–B zone border. At the T–B zone border, these activated T cells interact with cognate B cells, migrate into the germinal center and further differentiate into mature Tfh cells. In the absence of further interactions with an activated B cell, these nascent Tfh cells dissipate [45, 46]. Thus, signals from cognate B cells are necessary for the complete differentiation and maintenance of Tfh cells in the germinal center [20]. CD40 is a special molecule expressed on the surface of B cells, which can interact with the ligand CD40L (CD154) on T cells. The CD40 molecule and its ligand CD40L belong to the tumor necrosis factor (TNF) superfamily. The CD40–CD40L interaction is important in T–B cell crosstalk to induce an effective adaptive immune response. The CD40–CD40L signaling in B cells plays an important role in the generation and survival of long-lived plasma cells and memory B cells [47], the activation of Tfh cells, and the interaction of activated Tfh cells with B cells and antigen-presenting cells (APCs) [48]. In addition, it provides the necessary environment for the expression of the transcription factor BCL-6 for Tfh cell differentiation [49]. The lack of CD40L may cause damage to the T-cell-mediated immune function, the maturation of B cells, and the generation of antibodies and subtype switch [48]. On the other hand, the binding of CD40 molecules on B cells to CD40L could up-regulate the expression of ICOSL [50], which may also promote Tfh cell differentiation. The evidence above suggests that the CD40 molecule of B cells plays an important role in the enhancement of the humoral immune response. Furthermore, studies have shown that activation of the AGEs–RAGE pathway could up-regulate the expression level of CD40 on the surface of monocytes or DC [51]. The increased expression of CD40 in macrophages infiltrating around the nerve tissue of diabetic patients was also observed [52]. In our study, we analyzed the levels of MHCII, CD80, CD86, and CD40 on B cells in the control group and the group treated with AGEs in vitro. The results showed that the expression of CD40 was significantly increased in the AGEs group compared to the control group. However, there was no statistical difference in the expression of MHCII, CD80, and CD86. Thus, it is reasonable to speculate that the promotion of Tfh cells by AGEs might be attributed to the enhancement of CD40 expression on B cells.

Patients with diabetes may suffer infections at higher frequencies, suggesting that decreased host immunity to infections develops in patients with diabetes. NK cells are innate immune cells that can directly recognize and kill foreign, infected, and malignant cells and serve as a bridge between innate and adaptive immunity [53]. Growing evidence from mouse and human studies have shown that NK cells are also involved in the development or progression of autoimmune diseases, such as multiple sclerosis (MS) and systemic Lupus Erythematosus (SLE) [53–55]. It has been reported that NK cytotoxicity was impaired in patients with diabetes [56]. Our previous data showed that CXCR5^+^ NK cells were positively correlated with Tfh cells and might promote humoral immune response [57]. Our study showed that the percentages of NK cells in DM rat spleens were not different from control rats. However, CXCR5^+^ NK cells were upregulated in DM rats, which was consistent with the increase of Tfh cells. It is reported that in the peripheral blood of patients with type II diabetes, there was an increased ratio and activity of NKT cells [58]. In the study, we also found an increased percentage of NKT cells in the DM+EAMG group.

In conclusion, the present results showed STZ-induced EAMG displayed more severe weakness, due to the higher concentration of anti-R97–116 IgG antibody, more percentages of antibody-secreting cells and Tfh cells. Diabetes promoted B cell activation and upregulated the expression of CD80 and CD86, which may account for the differentiation of CD4^+^ T cells from Treg cells towards Th1/Th17 cells. In addition, diabetes enhanced NK function as well as NKT percentages. Finally, the effect of AGE on Tfh differentiation was verified and it is dependent on B cells in vitro. Overall, diabetes caused aggravation in EAMG rats through the enhancement of both innate and humoral immunity. Our study had several strengths. We found the effects of DM on EAMG rats and explored the mechanism. However, our study was limited by the absence of glucose of AGEs reduction, which may give an outlook and impulse for further research.

## Data Availability

The data sets used and/or analyzed during the current study are available from the corresponding author on reasonable request.

## Abbreviations

AGEs: Advanced glycation end products
APCs: Antigen-presenting cells
ASCs: Antibody‐secreting cells
BG: Blood glucose
CXCR5: C-X-C chemokine receptor 5
DCs: Dendritic cells
DM: Diabetes mellitus
GC: Germinal center
ICOS: Inducible co-stimulator
LOMG: Late-onset myasthenia gravis
LPS: Lipopolysaccharide
MG: Myasthenia gravis
MNCs: Mononuclear cells
NK cells: Natural killer cells
NKT cells: Natural killer T cells
RAGE: Advanced glycation end products
STZ: Streptozotocin
Tfh: Follicular helper T
Th: T helper cells
Trge cells: Regulatory T cells
