# Correction to: Diabetes mellitus exacerbates experimental autoimmune myasthenia gravis via modulating both adaptive and innate immunity

**DOI:** 10.1186/s12974-021-02363-0

**Published:** 2021-12-31

**Authors:** Peng Zhang, Chun-Lin Yang, Tong Du, Yu-Dong Liu, Meng-Ru Ge, Heng Li, Ru-Tao Liu, Cong-Cong Wang, Ying-Chun Dou, Rui-Sheng Duan

**Affiliations:** 1 Department of Neurology, The First Affiliated Hospital of Shandong First Medical University & Shandong Provincial Qianfoshan Hospital, No. 16766, Jingshi Road, Jinan, 250014 People’s Republic of China; 2 Shandong Institute of Neuroimmunology, Jinan, 250014 People’s Republic of China; 3 Shandong Key Laboratory for Rheumatic Disease and Translational Medicine, Jinan, 250014 People’s Republic of China; 4 Present Address: School of Medicine, Tongji University, Shanghai, 200092 People’s Republic of China; 5 College of Basic Medical Sciences, Shandong University of Traditional Chinese Medicine, Jinan, 250355 People’s Republic of China

## Correction to: J Neuroinflammation (2021) 18:244 https://doi.org/10.1186/s12974-021-02298-6

Following publication of the original article [1], the authors noticed that there was an error in the order of Figs. 3 and 4: the two figures were inadvertently transposed with one another. The original article has been updated and the correct version can be found in this erratum. (Figs. 3 and 4).

**Fig. 3:**
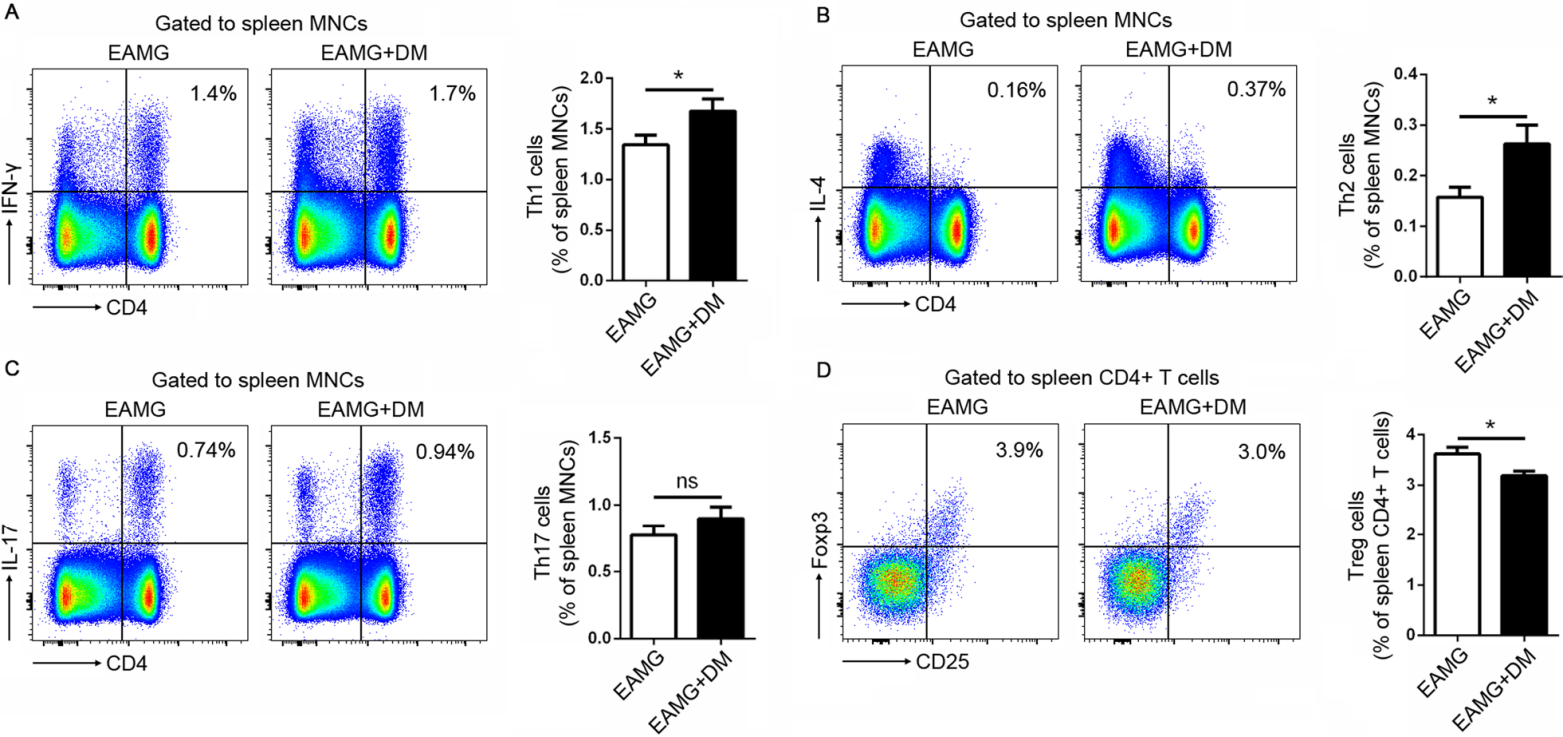
Effects of diabetes on the T helper cell differentiation in EAMG rats. The percentages of Th1 (**A**), Th2 (**B**), Th17 (**C**), and Treg cells (**D**) among spleen MNCs were analyzed by flow cytometry. Data were from two independent experiments and expressed as mean ± SEM. *n* = 8 in the DM + EAMG group and *n* = 7 in the EAMG group. The significance of differences was assessed by the Unpaired Student’s *t*-test. ns means not significant, *p < 0.05

**Fig. 4:**
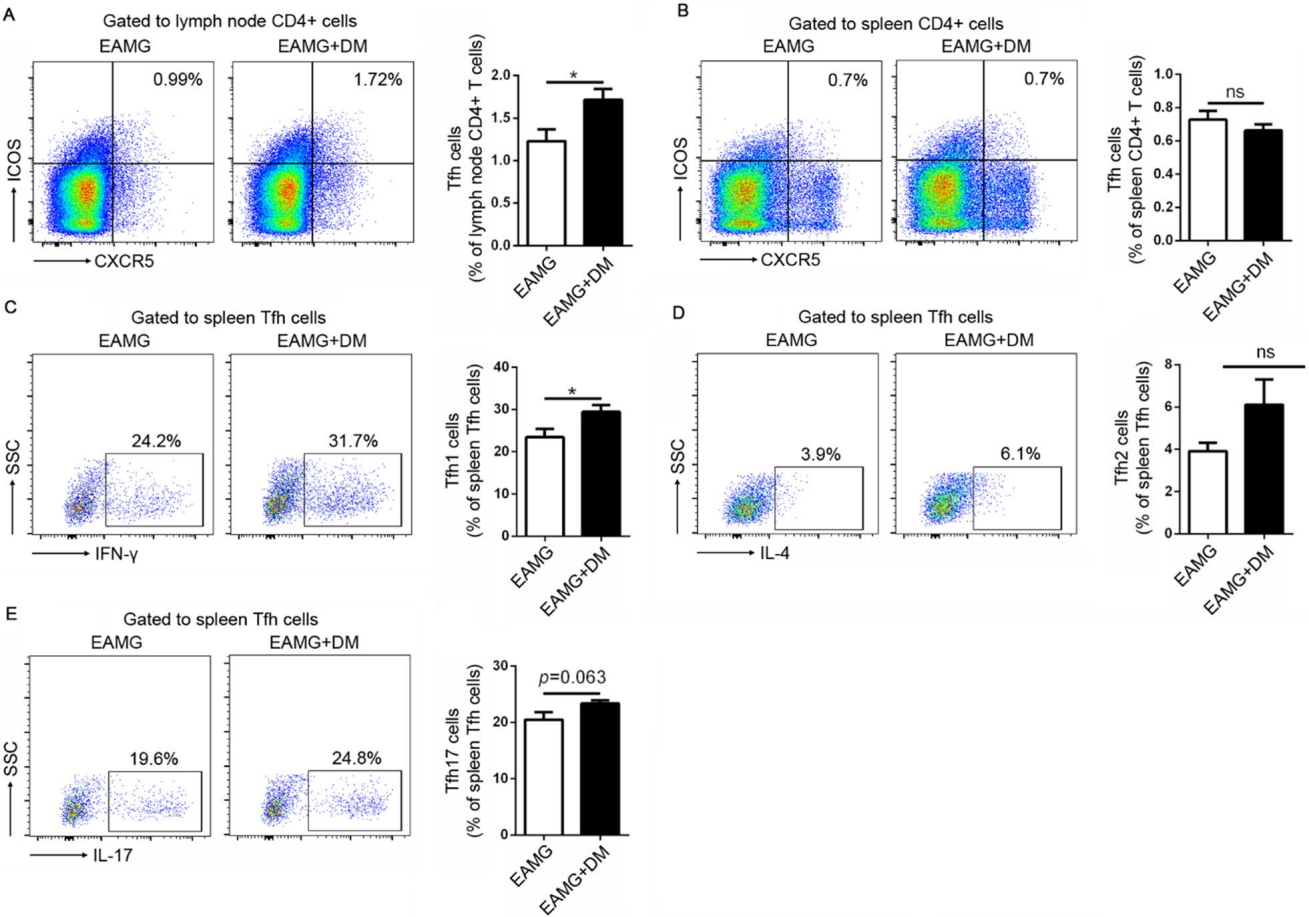
Effects of diabetes on Tfh cells and the subtypes. The percentages of Tfh cells in the lymph nodes were assessed (**A**). The percentages of total Tfh cells (**B**), Tfh1 (**C**), Tfh2 (**D**), and Tfh17 (**E**) among spleen MNCs were analyzed by flow cytometry. Data were from two independent experiments and expressed as mean ± SEM. *n* = 8 in the DM + EAMG group and *n* = 7 in the EAMG group. The significance of differences was assessed by the Unpaired Student’s *t*-test. ns means not significant, *p < 0.05

## References

[1] Zhang P, Yang C-L, Tong Du, Liu Y-D, Ge M-R, Li H, Liu R-T, Wang C-C, Dou Y-C, Duan R-S (2021). Diabetes mellitus exacerbates experimental autoimmune myasthenia gravis via modulating both adaptive and innate immunity. J Neuroinflamm.

